# Is Shame Managed Through Mind-Wandering?

**DOI:** 10.5964/ejop.v15i4.1787

**Published:** 2019-12-19

**Authors:** Neda Sedighimornani

**Affiliations:** a Department of Psychology, University of Bath, Bath, UK; University of Bari Aldo Moro, Bari, Italy

**Keywords:** shame, mind-wandering, pride, shame management, emotion

## Abstract

Shame is a notoriously unpleasant emotion, and although claims about the mechanisms through which we might manage it are none too scarce, relatively little empirical evidence is available concerning how people tend to cope with it. As such, the present study sought to investigate the effects of shame on mind-wandering. To do this, 120 participants were recruited and systematically assigned to one of the three groups, namely shame, pride, or control condition, and traits shame and self-compassion were measured for each participant. In order to assess the frequency of the incidents of mind-wandering, the participants were asked to recall a personal experience of shame or pride and then a reading task of few pages of geography followed. The duration participants spent on the reading task, their scores on a reading comprehension test, their self-reported frequency of mind-wandering, and their reported number of unrelated thoughts during the recall were used as a measure of mind-wandering. The results demonstrated that participants in the shame condition did not differ from those in the pride and control conditions in terms of mind-wandering. In spite of that, participants who had initially scored higher on trait shame (i.e., suffered from chronic shame) reported a significantly higher frequency of mind-wandering. This being the result, the underlying reasons for, and implications of, the findings were discussed.

As a human emotion, shame can be an unbearably painful and devastating experience. Put in an individual’s natural response to shame could range from aversion, to removal, to suppression, or even denial of the feeling of shame. The issue has attracted so much attention insomuch that the literature abounds with theories on how individuals cope with this natural, yet negative human emotion.

One such theory was carried out by Nathanson (1992) where he presented a shame management model. According to his model, individuals engage in four maladaptive strategies—i.e., avoidance, withdrawal, self-attack, and attacking other—in order to deal with their sense of shame. In another theory, Kaufman (1996) proposed that individuals resort to defensive strategies such as rage, contempt, humour, withdrawal, denial and blame of others as well as striving for perfection or power in order to cope with shame. Goss and Allan (2009) explored responses to shame in individuals with eating disorders and concluded that individuals adopt various coping strategies, such as aggression, submission, concealment, avoidance and withdrawal, destruction of the object of shame (e.g., self-harm), compensation (reparation), or help-seeking. More recently, Schoenleber and Berenbaum (2012) identified three classes of dysfunctional shame-regulation strategies: 1) prevention (e.g., perfectionism, fantasy); 2) escape (e.g., social withdrawal and the diversion of attention from self); and 3) aggression (either self-directed or other-directed).

To recapitulate briefly, theories, studies, and academic claims suggest that shame-based responses can range from avoidance and withdrawal, to attacking others, to striving for power and perfection. However, in support of these claims, there is a paucity of empirical evidence.

One exception, however, is a study by de Hooge, Zeelenberg, and Breugelman (2010, 2011). They showed that shame motivates individuals to seek self-enhancement strategies for the purpose of restoring their devalued self. Their findings showed that after experiences of shame, individuals engage in different hypothetical compensatory actions to improve their self-image. For example, to enhance their self-image, shamed individuals were more likely to undertake a difficult and challenging task than a simple or easy one which did not affect their self-image. Along similar lines, Chao, Cheng, and Chiou (2011) demonstrated that shamed participants spent more time on an unknowingly unsolvable problem than did the control group. The authors, thus, suggested that shamed participants were more determined to untie the problem and display their competence, which would in turn help them affirm a positive view of self.

Rather contrarily to the studies above, some studies suggest that individuals vulnerable to the experience of shame are inclined to use withdrawal or avoidance-based strategies (Sheikh & Jenoff-Bulman, 2010; Yi & Baumgartner, 2011). Reid, Harper, and Anderson (2009), for example, found that hypersexual patients react to the experience of shame with withdrawal or self-attack tendencies. Proneness to shame has also been associated with escapist responses (e.g., Tangney, 1995; Tangney, Miller, Flicker, & Barlow, 1996), anger or blame-shifting (Stuewig, Tangney, Heigel, Harty, & McCloskey, 2010; Thomaes, Stegge, Olthof, Bushman, & Nezlek, 2011), self-focused attention (Joireman, 2004), chronic rumination over the shameful incident (Orth, Berking, & Burkhardt, 2006), and alcohol and drug abuse (Dearing, Stuewig, & Tangney, 2005).

It is generally very difficult to talk about shameful experiences (Keltner & Buswell, 1996). Moreover, people’s descriptions of shame are very abstract and vague (Tangney, 1992). This perhaps shows that shamed individuals try to physically or emotionally distance themselves from shame-inducing experiences (Tangney & Dearing, 2002). They may avoid thinking about the incident, and, if reminded, they may distract themselves by thinking about something less intense and unpleasant, or they may change the focus of their attention from the shameful incident to something more neutral or positive.

Schoenleber and Berenbaum (2012) hypothesize that individuals attempt to disengage themselves mentally from their shameful experiences by engaging in daydreaming or mind-wandering. Although the relationship between daydreaming/mind-wandering and shame has not yet been empirically investigated, it is a common belief that daydreaming increases at times of stress (e.g., Greenwald & Harder, 1997) or that daydreaming may help those who have experienced failure (Lynn & Rhue, 1998).

Smallwood, O’Connor, Sudbery, and Obonsawin (2007) have defined mind-wandering as “a shift in the focus of attention away from the here and now towards one’s private thoughts and feelings” (p. 818). When the mind wanders, attention is diverted away or decoupled from the task at hand and is instead directed towards private thoughts or concerns. The occurrence of mind-wandering, therefore, impairs task performance (Smallwood, McSpadden, & Schooler, 2008). Studies have shown that when the mind wanders, reading comprehension, memory retrieval, information encoding, and signal detection are negatively affected (Smallwood et al., 2008; Smallwood & Schooler, 2006).

Not surprisingly, Mind-wandering and emotions also seem to be interrelated. This relationship, however, is relatively complex. Past research indicates that negative emotions lead to mind-wandering (Smallwood, Fitzgerald, Miles, & Phillips, 2009; Smallwood, O’Connor, & Heim, 2005). For example, dysphoric individuals seem to be more concerned with self-relevant information and thus report a higher level of off-topic thoughts compared to the general population (Smallwood et al., 2007); or dysphoric students’ ability to concentrate on different cognitive tasks, such as reading, proofreading, or listening to a lecture seems to be limited (Lyubomirsky, Karsi, & Zehm, 2003).

While investigating the association between mood and mind-wandering, Smallwood et al. (2009) found that participants who were induced with negative emotions reported a higher frequency of unrelated thoughts than those put in a positive mood. The researchers also reported that the participants in the negative mood group made more errors on a Sustained Attention to Response Task (SART). Occasionally used to indirectly measure mind-wandering, SART is a go/no-go task in which participants are asked to respond as quickly as possible to a non-target and to inhibit their responses to the target.

Studies also suggest that negative emotions are *antecedent* to mind-wandering. Mrazek, Smallwood, and Schooler (2012), for instance, demonstrated that when participants are exposed to a stereotypical threat that induces negative emotions (e.g., asking female participants to do a mathematics test in front of male participants), the frequency of recourse to mind-wandering rises. That said, it is equally possible that mind-wandering *precedes* negative emotions. Kilingsworth and Gilbert (2010) found that after experiencing mind-wandering, participants reported more negative emotions. It can thus be concluded that mind-wandering and the inability to curb it distressed the participants.

The results obtained from studies on mind-wandering and emotions have thus far been encouraging. It is assumed that this research area can be further explored through studies that address specific negative emotions, such as shame, or still by using different methods of measuring mind-wandering.

Similar to shame, pride is a self-conscious emotion. However, unlike to shame, pride is positively valenced (Tangney, 1995). According to Tracy and Robins (2007), authentic pride is elicited when individuals make internal, stable, and uncontrollable attributions. After feelings of pride, the self is elevated, strong, and accepted (Stanculescu, 2012). As pride is a positive emotion, the way it is managed has stimulated scant interests of researchers. Since shame is the main focus of this study, the researcher decided to bring in pride as, this time, a positive self-conscious emotion whose characteristics contradict those of shame. This would allow the researcher to control the effects of emotional conditions and to examine whether a negative self-conscious emotion such as shame would affect mind-wandering differently from a positive self-conscious emotion such as pride. Further to that, research on mind-wandering and pride can also—however indirectly—contribute to the literature on pride.

On the basis of this brief overview, the current study was set to investigate the relationship between mind-wandering and shame and hypothesised that shame had a significant effect on mind-wandering. More specifically, it was hypothesised that:

1. negative moods increase the likelihood of mind-wandering about a past event and ruminating on it (Smallwood & O’Connor, 2011). Therefore, feelings of shame may lead individuals to ruminate over the shameful incident and the past;
2. individuals may engage in mind-wandering in order to improve their mood (Mason, Brown, Mar, & Smallwood, 2013);
3. shamed individuals are likely to use different strategies to avoid thinking about or feeling shame. They may thus engage in mind-wandering/daydreaming in order to escape painful feelings of shame; and

Given the experimental nature of the present study and the induction of a state of shame (v. pride), it was thought it would be necessary to measure, in relation to mind-wandering, both trait shame and trait self-compassion. This would allow to explore how high-scoring individuals in these measures differed from those who obtained lower scores.

## Method

### Participants

For the purposes of this study, a total of 120 (*N* = 120) participants (82 females and 38 males, *M*_age_ = 24.28, *SD* = 8.12) were recruited using convenient sampling. Next, each participant was, on the premise of systematic sampling, assigned to one of the three groups of shame (*n* = 40, 13 males and 27 females, *M*_age_ = 25, *SD* = 7.37), pride (*n* = 40,12 males and 28 females, *M*_age_ = 24.47, *SD* = 9.03), or control (*n* = 40, 13 males and 27 females, *M*_age_ = 23.35, *SD* = 8.27) in that the first participant was assigned to group shame, the second to pride, the third to control, and so forth, putting an equal number of 40 participants in each group to test.

The sample included students from the University of Bath (60%), the university staff, and visitors to the campus at the time of the study. The majority of the participants (93.3%) were native English speakers and the remainder were adequately proficient in English for the purpose of this study.

### Procedure

The study was first piloted with a group of approximately 20–30 volunteers. The preliminary results and the comprehensive feedback from theses participants were then used in order to improve on the procedure.

In order to recruit participants for the main study, it was advertised at the author’s university of affiliation. Volunteers were given the choice to choose between a remuneration of either £5 or course credits to be awarded if they took part. The study was conducted at the laboratory of social psychology at the University of Bath and was approved by the Department of Psychology Research Ethics Committee (Reference number: 12–105).

In order to respect privacy and preserve anonymity and confidentiality, only one participant at a time was invited to the laboratory. All the materials were presented on a computer screen. The responses of the participants on the computer were then recorded using a piece of experimental psychology software called Media Lab which would allow to measure response time and word count of the writing tasks.

At the end of the experiment, the participants were fully debriefed and thanked.

### Measures

#### Recall and Writing Task for Inducing Shame and Pride

Emotions were evoked using an autobiographical recall procedure (Yang, Yang, & Chiou, 2010). The participants in the shame and pride groups were first asked to take a few seconds and think about shame or pride in general. They were then asked to recall and write, as vividly as possible, about a personal experience when they had felt a spurt of shame or pride. Next, by eliciting the responses to the following questions, they were invited to think about the incident in more detail: 1) What was the emotional event? 2) Why did it happen? 3) How did you feel then? and 4) What was the consequence of that event? (Yang et al., 2010). Most importantly, they were asked to recall how they felt, re-evoking the feelings they had at the time.

With the aim replicating, as much as practicable, the experimental conditions, the participants in the control group were first asked to recall and write about what they had eaten for dinner the night before. More specifically, they were asked to think and write about the following questions: 1) What did you have last night for dinner? 2) Why did you have that? 3) How did you prepare that (or where did you buy that)? and 4) How long did it take to prepare (or buy) that meal?

#### Manipulation Check

To see whether variation in the manipulated variables cause any differences, all participants were asked to indicate the extent to which they felt shame, pride, sadness, or happiness after writing about their experiences. They would then rate each of these feelings on a scale of 1 to 5, where 1 is *did not have such a feeling at all* and 5 is *extremely felt it*.

#### Frequency of Mind-Wandering

In order to measure mind-wandering, all participants were instructed to read a none-too-engaging three-page long text about the geography of rivers. They then answered the 10-point scale question of “To what extent did your mind wander during the reading?” This would be an indication of the frequency at which a given participant’s mind wandered.

#### Comprehension Test

Since mind-wandering is thought to affect text comprehension as well as reading speed (Sayette, Reichle, & Schooler, 2009), participants were also asked to answer six “surprise” comprehension questions about the text. Higher scores in the comprehension test indicated better comprehension and thus less mind-wandering.

#### Retrospection of Unrelated Thoughts

Retrospection was also used as a measure of mind-wandering. Having read the text used in the comprehension test, participants were asked to report on their unrelated thoughts while reading.

#### Time Spent on Each Task

Time spent on each task, including among others recalling, writing and reading, was measured up to the millisecond using the specialised psychology software, Media Lab.

#### Wordcount

The number of words on all writing tasks was calculated by Media Lab.

#### Frequency of Emotionally-Related Thoughts, Intensity of Emotional Experience, and Level of Avoidance

After the reading task, the participants in the pride and shame conditions were given a 5-point Likert-scale (1 = least; 5 = most) with three questions which measured 1) frequency of emotionally-related thoughts, 2) intensity of their emotional experience, and 3) their level of avoidance.

The three questions were: 1) “During the reading task, to what extent did you have thoughts or feelings related to the memory (shame or pride) we asked you to think/write about?” 2) “To what extent did you feel shame (or pride for those assigned to the pride condition) when you were thinking and writing about your personal experience of shame (or pride)?” and 3) “Sometimes strong emotions can be uncomfortable for us. Our minds are reluctant to stay with them. To what extent did you find that, during the recalling and writing stages, your mind would drift off to different things so as not to feel a strong emotion?”

#### Trait Shame and Self-Compassion

At the end of the experiment, the participants were asked to complete the trait shame and self-compassion scales.

Trait shame was measured using the Experience of Shame Scale (ESS) (Andrews, Qian, & Valentine, 2002). This scale consists of 25 items that ask direct questions about three specific characteristics (i.e., personal traits, behaviour, and physical appearance) about which individuals may feel shame. Each respondent should rate each of the items on the basis of a 4-point scale (1 =*not at all*, 4 = *very much*), where a higher score indicates a higher level of shame. The internal consistency for this scale has been reported as .92 (Robins, Noftle, & Tracy, 2007).

Self-compassion was assessed with the Short Form of the Self-Compassion Scale (SCF-SF) (Raes, Pommier, Neff, & Van Gucht, 2010). This scale consists of 12 items rated on a 5-point scale (1= *almost never*, 5= *almost always*), where a higher score indicates a higher level of self-compassion. An estimated Cronbach’s alpha for this scale is above .86 (Raes et al., 2010).

### Data Analysis

A one-way analysis of variance (ANOVA) was used to determine whether the differences between the means for conditions were significant. More specifically, ANOVA was carried out to determine how independent variables, that is, the experimental conditions of shame, pride, and control, affected the dependent variables of manipulation check, frequency of mind-wandering, comprehension test score, number of unrelated thoughts and words, time spent on each of the tasks, and wordcount. In addition, to further predict the effect of the traits self-compassion and shame on the frequency of mind-wandering, two linear regression analyses were conducted.

## Results

### The Experience of Shame Scale (ESS) and the Self-Compassion Scale (SCF-SF)

The internal reliabilities for the Self-Compassion Scale (α = .84) and the ESS (α = .92) were relatively high. The mean self-compassion score was 34.12 (*SD* = 7.31), and the mean for the ESS was 57.45 (*SD* = 13.59). The correlation between self-compassion and shame was −.55 (*p* < .001).

### Emotion Manipulation Check

The shame induction protocol was successful, with participants assigned to the shame condition feeling significantly more shame (*M* = 2.88, *SD* = 1.02) than those assigned to the pride (*M* = 1.43, *SD* = 0.78) and control condition (*M* = 1.50, *SD* = 1.50) (*F* (2, 117) = 34.60, *p* < .001). The participants in the shame group also felt significantly more sadness (*M* = 2.38, *SD* = 1.25) than the participants assigned to either the pride condition (*M* = 1.68, *SD* = 1.07) or the control condition (*M* = 1.55, *SD* = 0.85) (*F* (2, 117) = 6.90, *p* < .001). Participants in the shame condition reported a significant level of shame after controlling for sadness (*F* (2, 116) = 25.13, *p* < .001).

The pride induction protocol was also successful. Participants assigned to the pride condition felt significantly more pride (*M* = 3.60, *SD* = 1.06) than those assigned to either the shame (*M* = 1.73, *SD* = 1.06) or the control condition (*M* = 2, *SD* = 0.96) (*F* (2, 117) = 38.83, *p* < .001). In addition, participants assigned to the pride condition reported a significantly higher level of happiness (*M* = 3.40, *SD* = 0.90) than those assigned to either shame (*M* = 1.85, *SD* = 0.97) or control (*M* = 2.65, *SD* = 0.74) conditions (*F* (2, 117) = 24.03, *p* < .001). Along similar lines with the shame group, the participants in the pride condition reported a significant level of pride after controlling for happiness (*F* (2, 116) = 15.71, *p* < .001).

### Wordcount of Written Tasks

Participants assigned to the shame condition generally wrote longer descriptions of their personal experiences. That said, a one-way ANOVA procedure on the hard data (see Table 1) yielded no significant intergroup differences with respect to the length of the written tasks.

**Table 1:** Mean, Standard Deviation, and ANOVA Results for Variables in Shame, Pride, and Control Conditions

Variable	Shame		Pride		Control		ANOVA	
	*M*	*SD*	*M*	*SD*	*M*	*SD*	*F*	*p*
Wordcount	157.05	84.60	139.87	80.45	131.30	71.70	1.10	.34
Recall time	679,393	41,066	50,333	24,109	39,335	13,318	10.48	<.01
Describe time	432,563	226,189	374,543	215,321	290,362	170,181	4.98	<.05
Mind-wandering	5.75	2.35	5.90	2.42	5.85	2.51	0.04	.96
Comprehension test	3.55	1.39	3.07	1.56	3.57	1.55	1.40	.25
Unrelated thoughts	2.60	2.28	2.90	2.15	3.12	2.06	0.57	.57
Unrelated words	48.22	51.86	49.42	45.63	46.30	36.48	0*.*05	.95
Reading time	100,407	40,730	92,446	44,454	91,074	29,348	0.66	.52

*Note*. Mind-wandering = frequency of occurrences of mind-wandering; Comprehension test = scores in the reading test; Unrelated thoughts = numbers of unrelated thoughts that participants had while they were reading the text; Unrelated words = number of words written in response to the unrelated thought question; Reading time = time participants spent on the reading task; ANOVA = analysis of variance; Time values were recorded in milliseconds.

### Recall and Describe Time

A one-way ANOVA on the recall and describe time values demonstrated that participants assigned to the shame condition spent significantly more time recalling and describing their personal experiences of shame than the participants assigned to either pride or control condition (Table 1). However, participants in the three conditions did not differ significantly in terms of the frequency of mind-wandering, performance on the comprehension test, or number of unrelated thoughts.

### Trait Shame, Self-compassion and Mind-Wandering

Two regression analyses were conducted to examine the influence of trait self-compassion and shame on mind-wandering. It was found that trait self-compassion did not significantly predict the frequency of mind-wandering (β = −.16, *p* = .06). Nevertheless, regardless of the experimental condition, trait shame significantly predicted the frequency of mind-wandering (β = .21, *R* ^2^ = 0.04, *p* < .05).

### Frequency of Emotional Thoughts, Intensity of Emotion, and Level of Avoidance

With respect to intensity of emotional experience, participants assigned to pride condition felt significantly more pride and were more able to evoke feelings of pride when they were thinking and writing about their personal experiences of pride than participants who were assigned to the shame condition and who were asked to recall a personal experience of shame (Table 2).

**Table 2:** Mean and Standard Deviation for Emotional Thoughts, Intensity, and Avoidance in Shame and Pride Conditions

Variable	Shame		Pride		*t*-test	
	*M*	*SD*	*M*	*SD*	*t*	*p*
Emotional thoughts	3.80	2.45	3.43	2.77	0.64	.52
Intensity	6.85	2.71	8.13	2.32	−2.28	<.05
Avoidance	4.08	2.45	4.03	2.68	0.08	.93

*Note. SD* = standard deviation; Emotional thoughts = extent to which participants had thoughts and feelings related to the memory during the reading task (Q1); Intensity = extent to which participants felt shame/pride when they were recalling their experience (Q2); Avoidance = extent to which participants’ mind wandered so as to avoid feeling a strong emotion (Q3).

### Effects of Trait Shame and Self-Compassion on Emotional Thoughts, Intensity of Emotional Experience, and Avoidance

It was decided to next consider the effects of trait shame and self-compassion on the frequency of *emotional thoughts*, the *intensity of emotional experience*, and *avoidance* after manipulating shame and pride. Trait shame did not significantly predict the level of emotionally related thoughts during the reading task (Question 1), the intensity of emotional experience (Question 2), or the level of avoidance (Question 3) for either condition (Table 3).

**Table 3:** Regression Analyses Predicting Emotional Thoughts, Intensity, and Avoidance From Trait Shame and Self-Compassion in Shame and Pride Conditions

Variable	Shame Condition						Pride Condition					
	Emotional Thoughts		Intensity		Avoidance		Emotional Thoughts		Intensity		Avoidance	
	β	*p*	*Β*	*p*	β	*p*	β	*p*	*Β*	*p*	β	*p*
Trait shame	.08	.60	.16	.30	−.04	.78	−.01	.97	.09	.57	.23	.14
Self-compassion	−.20	.19	−.17	.27	−.12	.46	−.02	.89	.09	.56	−.44	.004

*Note.* β *=* Standardised Coefficients; Emotional thoughts = extent to which participants had thoughts and feelings related to the memory during the reading task (Q1); Intensity = extent to which participants felt shame/pride when they were recalling their experience (Q2); Avoidance = extent to which participants mind wandered in order to avoid feeling a strong emotion (Q3).

Trait self-compassion did not significantly predict the level of emotionally related thoughts or the intensity of emotional experience in shame and pride conditions, either.

For the shame condition, trait self-compassion did not explain a significant proportion of the variance in the level of avoidance. However, for the pride condition, trait self-compassion significantly predicted the extent of avoidance (Table 3).

The findings indicated that participants in the pride condition who had a higher score for self-compassion were less likely to avoid their evoked emotions, while those who had obtained a lower score on self-compassion were reluctant to stay with their feelings of pride and were rather inclined to avoid these feelings.

## Discussion

The study was novel by considering mind-wandering as a method of shame management. Throughout the literature, there are many theories about how shame might be dealt with; however, relatively little empirical evidence is available. The main aim of this study was to examine whether after the experience of shame individuals engage in mind-wandering. This adds to the literature by opening a discussion regarding this issue. Further, it examines one of the long standing claims in the literature.

This study showed that participants in the shame condition spent significantly more time recalling and writing about their shameful experiences than participants in either the pride or control condition. These results are consistent with previous studies (cf. Tangney, 1992) indicating that retrieval of shame-related memories can be a taxing task. In fact, this study demonstrates that participants in the pride condition were more able to evoke feelings of pride in comparison to those who were in the shame condition. It seems that evoking feelings of pride is considerably less demanding than evoking feelings of shame.

As for mind-wandering, the results indicated that across all experimental groups, individuals high in trait shame reported a significantly higher frequency of mind-wandering than participants low in trait shame. This finding implies that individuals suffering from significant levels of shame engage in more mind-wandering than those who do not experience shame often which is consistent with the idea of managing trait shame through mentally disengagement (Schoenleber & Berenbaum, 2012).

Excluding the association between trait shame and the frequency of mind-wandering, the findings of this study indicate that shame does not have a significant effect on mind-wandering. Moreover, participants in the shame condition did not have a significantly lower (or higher) score in the comprehension test; they did not spend significantly more time on the reading task; nor did they report a higher number of unrelated thoughts during the reading task than participants in the pride or control conditions. These results, therefore, translate into the rejection of the main hypothesis of the present study.

In the pride condition, however, self-compassion was inversely associated with level of avoidance; it significantly predicted avoidance of emotional thoughts and feelings during the reading task. Such association and prediction, notwithstanding, were not observed in the shame condition. In addition, self-compassion did not influence the frequency and intensity of emotional thoughts and feelings in the shame condition, a result which is inconsistent with the findings previously reached in the literature. For example, in the literature on self-compassion, Leary, Tate, Adams, Allen, and Hancock (2007) reported that self-compassion functions as a buffer against negative emotions and life events and that self-compassionate individuals seemed to be less affected by failures and negative outcomes (Leary et al., 2007). This relationship is yet to be further probed into in future studies.

In terms of the lack of a clear causal relationship between shame and mind-wandering, several factors need be considered:

First, it is debatable whether the methods used to assess mind-wandering can detect the associations between negative emotions and mind-wandering. For example, Mrazek et al. (2012) found that negative emotion was correlated with errors on the Sustained Attention to Response Task (SART), but not with a self-reported measure of daydreaming. They then concluded that “[t]he association between mind-wandering and negative effect may emerge only during pronounced task-disengagement” (Mrazek et al., 2012, p. 5).

Second, and more importantly, shame can be a transitory emotional state (state shame) or chronic emotional disorder (trait shame). Shame as state emotion is likely to last for only a fleeting period. It may also be substituted by a less painful emotional state, such as anger or regret (Hahn, 2000). Individuals also cope with state shame or trait shame differently (Behrendt & Ben-Ari, 2012; Lickel, Kushlev, Savalei, Matta, & Schmader, 2014). For instance, de Hooge et al. (2010, 2011) challenged the link between state shame and withdrawal tendencies, arguing that individuals who experience state shame attempt to repair a devalued self rather than withdraw from the situation. According to this argument, shame-participants were expected to have outperformed the control group in the reading and memory tasks in this study; nonetheless, the inter-condition differences were non-significant.

Lickel et al. (2014) also demonstrated that after recalling a personal experience of shame, participants reported a higher degree of motivation to change the self, describing an “urge to become a better person” or a strong desire to “change aspects of my personality” (p. 1052), in comparison to the participants who recalled a personal experience of embarrassment. However, according to Lickel et al. (2014), a strong determination to suppress painful feelings of shame and avoid shame inducing situations might counteract that desire for change.

In contrast, when the experience of shame looms frequently (i.e., chronic shame), and when individuals do not have access to resources or options to seek self-enhancement, they are likely to use avoidance-oriented strategies (de Hooge et al., 2010, 2011). For example, if one feels ashamed of being poor or unattractive, and does not have any option to change the situation, he or she may attempt to use escapism strategies (e.g., daydreaming/mind-wandering) in order to alleviate his or her mood or, in extreme situations, make life more bearable. This compares well with watching television or reading fiction, activities that act like solace, helping an individual forget the harsh reality of life. It is, therefore, possible that highly ashamed individuals (trait shame) engage in mind-wandering or daydreaming frequently and compulsively. The use of strategies like mind-wandering, however, may temper a negative mood and depression (Schoenleber & Berenbaum, 2012).

Nonetheless, it should be noted that using qualitative methods, Van Vliet (2008) concluded that individuals try to rebuild the self through five processes after experiencing shame: 1) connection: trying to socialise with others, talk about their shame experiences, or connect with a higher power; 2) refocusing: focusing on positive things, self-enhancement, and taking some action; 3) acceptance: accepting the shaming situation and expressing one’s feelings; 4) understanding external factors: separating the self from shame, developing self-awareness, and trying to evolve; and 5) resisting: rejecting negative judgements and challenging others’ beliefs about the self. Chen, Hewitt, and Flett (2015) similarly found a significant association between need to belong and shame; Leeming and Boyle (2013) found that connections with others and social validation were essential for rehabilitating feelings of shame.

The above research seems to imply that social relationships, social support, and a sense of belonging are essential for overcoming feelings of shame. However, a tendency to avoid or withdraw, which is often observed in individuals vulnerable to the experience of shame (Tangney, 1995), becomes a significant barrier to building social connections and relationships (Black, Curran, & Dyer, 2013).

### Limitations

The extent to which participants were able to evoke feelings of shame in the laboratory remains questionable. Participants might remember the shameful incident rather than the actual experience of shame; they may have come to terms with previous feelings or the situation may have been resolved, but it was still in their memory. In addition, writing about positive (Burton & King, 2004), negative, or stressful experiences and emotions (Baikie & Wilhelm, 2005) can have therapeutic effects; it may assist individuals to rectify their unresolved feelings. Therefore, recalling and describing a negative emotion may have contradictory effects. On the one hand, it may induce negative emotions, and on the other, it might resolve the negative emotions that had been evoked. This paradoxical effect of the autobiographical procedure, therefore, should well be acknowledged.

In addition, since the study relied on autobiographical recall to elicit shame, there was a great range of variations in shame memories and descriptions. Some participants recalled a situation where they had cheated on their partner, others described their academic failures, and still others recalled situations where they had done something wrong, such as lying, stealing or upsetting someone. Though autobiographical recall is a well-established methodology, it cannot ensure that only feelings of shame be evoked during the recall of the majority of these situations. The research on state shame indicates that individuals may behave less consistently after experiencing state shame (induced shame). Many factors can interfere with how people react to the experience of shame. Sometimes, it seems impossible to identify a distinct pattern. For example, factors such as situational context (Silfver, 2007), personality traits (Carver & Conner-Smith, 2010), and cultural factors (Bagozzi, Verbeke, & Gavino, 2003) may affect how individuals non-prone to shame cope/deal with it.

### Future Directions

Apparently, inducing shame in the laboratory is a very challenging task. It is still unclear whether shame can be studied in laboratory settings or to what extent it can be credible. Furthermore, there is a disparity between experiencing shame (state shame) and having a shame-prone identity (Harper, 2011). It is reasonable to believe that shame-prone individuals tend to use avoidance-oriented coping strategies (e.g., denial, mental disengagement, self-distraction, etc.) and that dealing with the experience of shame (state shame) depends more on the nature of the shameful event or the individuals’ disposition. Although continuing this line of research is laborious, it is likely that it can make a considerable contribution to our understanding of shame and coping strategies. Clearly enough, some coping strategies are more efficacious in promoting well-being than others.

As for future studies, it is important to understand the short-term benefits and long-term costs of the shame management methods. (Negative) mind-wandering might cause unhappiness and worry and may lead to rumination. Moreover, the empirical evidence also suggests that even those who gain pleasure from mind-wandering still feel distress when they notice that their mind has drifted and, thus, respond negatively (Mason et al., 2013). Since compulsive mind-wandering may occur automatically, unintentionally, or extemporaneously, it might be difficult to regulate. That is perhaps why mindfulness and the ability to train the mind to be present-oriented can be highly effective in regulating emotions and thoughts. Hence, for the future, it is necessary to direct the focus towards mind-wandering as a way of emotion management.
